# The Fission Yeast Homeodomain Protein Yox1p Binds to MBF and Confines MBF-Dependent Cell-Cycle Transcription to G1-S via Negative Feedback

**DOI:** 10.1371/journal.pgen.1000626

**Published:** 2009-08-28

**Authors:** Sofia Aligianni, Daniel H. Lackner, Steffi Klier, Gabriella Rustici, Brian T. Wilhelm, Samuel Marguerat, Sandra Codlin, Alvis Brazma, Robertus A. M. de Bruin, Jürg Bähler

**Affiliations:** 1 Department of Genetics, Evolution & Environment and UCL Cancer Institute, University College London, London, United Kingdom; 2 MRC Laboratory for Molecular Cell Biology, University College London, London, United Kingdom; 3 EMBL Outstation–Hinxton, European Bioinformatics Institute, Cambridge, United Kingdom; Yale University, United States of America

## Abstract

The regulation of the G1- to S-phase transition is critical for cell-cycle progression. This transition is driven by a transient transcriptional wave regulated by transcription factor complexes termed MBF/SBF in yeast and E2F-DP in mammals. Here we apply genomic, genetic, and biochemical approaches to show that the Yox1p homeodomain protein of fission yeast plays a critical role in confining MBF-dependent transcription to the G1/S transition of the cell cycle. The *yox1* gene is an MBF target, and Yox1p accumulates and preferentially binds to MBF-regulated promoters, via the MBF components Res2p and Nrm1p, when they are transcriptionally repressed during the cell cycle. Deletion of *yox1* results in constitutively high transcription of MBF target genes and loss of their cell cycle–regulated expression, similar to deletion of *nrm1*. Genome-wide location analyses of Yox1p and the MBF component Cdc10p reveal dozens of genes whose promoters are bound by both factors, including their own genes and histone genes. In addition, Cdc10p shows promiscuous binding to other sites, most notably close to replication origins. This study establishes Yox1p as a new regulatory MBF component in fission yeast, which is transcriptionally induced by MBF and in turn inhibits MBF-dependent transcription. Yox1p may function together with Nrm1p to confine MBF-dependent transcription to the G1/S transition of the cell cycle via negative feedback. Compared to the orthologous budding yeast Yox1p, which indirectly functions in a negative feedback loop for cell-cycle transcription, similarities but also notable differences in the wiring of the regulatory circuits are evident.

## Author Summary

Cells proliferate by growth and division, which is supported by different gene groups that are periodically induced at specific times when they are required during the cell cycle. These genes not only need to be induced at the right time but also repressed when they are no longer required; mistakes in gene regulation can lead to problems in cell proliferation and diseases such as cancer. A well-known regulatory complex functions just before cells replicate their DNA to induce genes required for this important transition. We show that in fission yeast this regulatory complex (MBF) induces a gene whose encoded protein (Yox1p) in turn binds to MBF and represses MBF-regulated genes. In the absence of Yox1p, the MBF-regulated genes do not fluctuate during the cell cycle but remain constantly induced. Thus, MBF sets up not only the induction but also the timely repression of its target genes via Yox1p. We also provide a global analysis of all the genes regulated by Yox1p and MBF. Together, our data uncover a new negative control loop, further highlighting the sophistication of gene regulation during the cell cycle, and illustrating regulatory similarities and differences between organisms.

## Introduction

Transcript levels of many genes fluctuate periodically as a function of cell growth and division, peaking at specific phases of each cell cycle. Such cell cycle-regulated gene expression seems to be a universal feature of proliferating cells [1]–[3]. The best characterized transcriptional wave is induced during the G1/S transition, named ‘Start’ in yeast and ‘restriction point’ in mammalian cells, when cells commit to DNA replication and thus to a new cell-division cycle. In mammalian cells, E2F-DP complexes control G1/S transcription and are deregulated in most cancer cells, highlighting the importance of this regulation [4]–[6]. In the fission yeast, *Schizosaccharomyces pombe*, a transcription factor complex termed MBF (MluI Cell Cycle Box [MCB] Binding Factor) represents the functional equivalent of E2F-DP. MBF activates transcription during the G1/S transition, binding to MCB promoter elements that are conserved from yeasts to humans. The MBF complex contains Cdc10p and at least two related ankyrin-repeat DNA-binding proteins, Res1p and Res2p [7]–[10]. Multiple studies have identified over 20 putative MBF target genes with roles in DNA replication, DNA repair, and cell-cycle control (e.g., [11]–[14]).

The MBF-dependent transcriptional program in fission yeast is subject to several regulatory inputs in response to both cell-cycle and external signals. Res1p and Res2p play critical, but poorly understood roles in MBF-dependent transcriptional regulation during the cell cycle [9],[15]. An additional factor, Rep2p, is crucial for MBF complex transcriptional activity [15]–[18]. Two negative regulatory circuits are known that ensure timely repression of MBF-dependent genes: the cyclin Cig2p inhibits MBF activity in G2-phase by phosphorylating Res1p [19], and Nrm1p acts as a transcriptional co-repressor for MBF-dependent genes [20],[21]. Both *cig2* and *nrm1* are regulated by MBF and are therefore involved in negative feedback loops. Here, we establish an additional regulatory factor, the homeodomain protein Yox1p, as a novel component of MBF that inhibits MBF-dependent transcription via negative feedback and that is essential for cell-cycle regulation of MBF target genes. We also report the target genes directly bound by Cdc10p and Yox1p at a genome-wide level.

## Results

**Figure 1:**
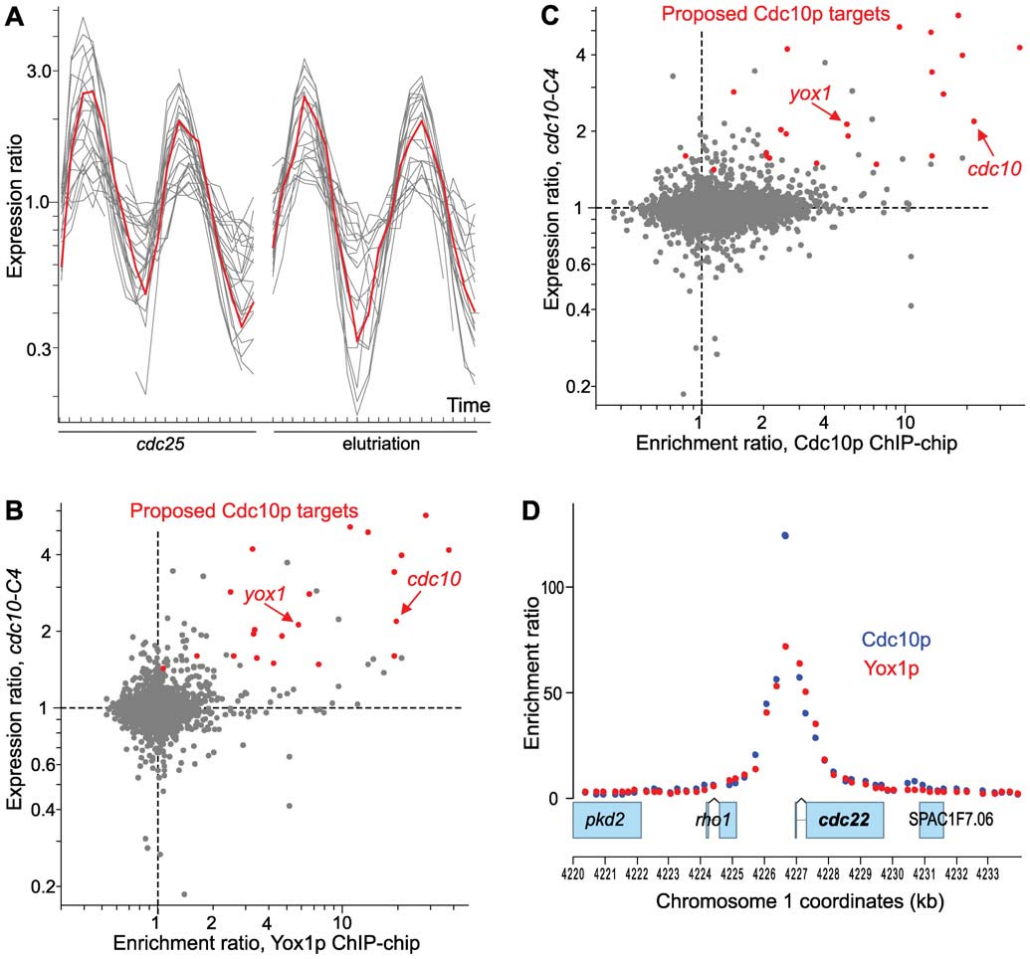
Yox1p is an MBF target and binds to MBF target genes. (A) *yox1* is periodically transcribed in the same cluster as MBF target genes. Two cell-cycle timecourse experiments of cells synchronized by *cdc25* block-release (left) or centrifugal elutriation (right) showing the expression profiles of 24 putative Cdc10p-regulated genes (data from [14]). The *yox1* expression profiles are shown in red. (B) Scatter plot showing gene expression ratios relative to wild-type in *cdc10-C4* mutant cells (which shows increased expression of MBF target genes [48]; mean data from four repeats [14]) *versus* the enrichment ratios upstream of genes in Yox1p ChIP-chip experiments (mean data from three repeats). The putative Cdc10p-regulated genes shown in (A) are highlighted in red, and the *cdc10* and *yox1* genes are indicated with arrows. (C) Scatter plot as in (B) but showing gene expression ratios relative to wild-type in *cdc10-C4* mutant cells *versus* the enrichment ratios upstream of genes in Cdc10p ChIP-chip experiments (mean data from three repeats). (D) The cell cycle-regulated gene *cdc22* [29] shows a particularly strong enrichment of both Cdc10p (blue) and Yox1p (red) upstream of its open reading frame. Example data from one repeat each are shown. Transcription of the four genes on chromosome 1 is from left to right (forward strand), and chromosome coordinates are indicated in kb.

The gene *SPBC21B10.13c* encodes a predicted transcriptional regulator that contains a homeobox domain. It is periodically expressed during S-phase (Figure 1A) [14] and is induced in response to ionizing radiation [22], which affects cell-cycle progression, and in response to hydroxyurea [14],[23],[24], suggesting a possible regulation by MBF. The protein encoded by this gene is similar to the budding yeast *Saccharomyces cerevisiae* homeodomain protein Yox1p (44.1% identity over 59 amino acids, BLAST score 128, E-value 5e-08), which is a cell-cycle transcription factor activated by the MBF-related SBF complex in budding yeast [25]–[27]. Due to functional similarities described below, we also named the *S. pombe* protein Yox1p (for ‘yeast homeobox’).

### *yox1* Is an MBF Target and Yox1p Binds to MBF-Regulated Promoters

Given that Yox1p is a predicted transcriptional regulator and a putative target of MBF, we analyzed the genome-wide binding sites of Yox1p. We performed chromatin immunoprecipitations combined with microarrays (ChIP-chip) [28] using a *yox1-HA* strain, an antibody against the HA epitope, and an Agilent genomic tiling array of ∼300 bp resolution. Among 24 genes proposed to be MBF targets (Figure 1A) [14], the promoters of 19 genes were substantially enriched in the Yox1p ChIPs, including *yox1* itself (Figure 1B). Among the remaining putative MBF targets, the promoters of two genes were not enriched (*ctp1* and *rep2*), while the promoters of three uncharacterized genes were not represented on the array (*SPAC17H9.18c*, and two members of repeated protein families: *SPBPB2B2.19c* and *SPAC750.05c*). These data suggest that Yox1p binds to MBF-regulated genes.

To confirm that Yox1p and MBF-regulated genes overlap and to gain more direct insight into the genome-wide binding sites of MBF, we performed ChIP-chip using an antibody against the MBF component Cdc10p. As for Yox1p, among the 24 proposed MBF targets, the promoters of the same 19 genes, including *cdc10* itself and *yox1*, were substantially enriched in the Cdc10p ChIPs (Figure 1C); the promoters of *rep2* and *ctp1* were not or only marginally enriched, respectively, and we could not obtain data for the same three genes indicated above for Yox1p. The peaks of enrichments for both Cdc10p and Yox1p were located just upstream of the open reading frames of the target genes, and the binding sites of these two factors could not be separated given the limited resolution of the tiling arrays (Figure 1D). We conclude that both Cdc10p and Yox1p bind to most promoters of the previously proposed MBF target genes, including their own genes. Taken together, these data show that *yox1* is transcriptionally activated by MBF, and Yox1p in turn binds itself to MBF target genes.

### Yox1p Dynamically Binds to Repressed MBF Target Promoters via MBF

**Figure 2:**
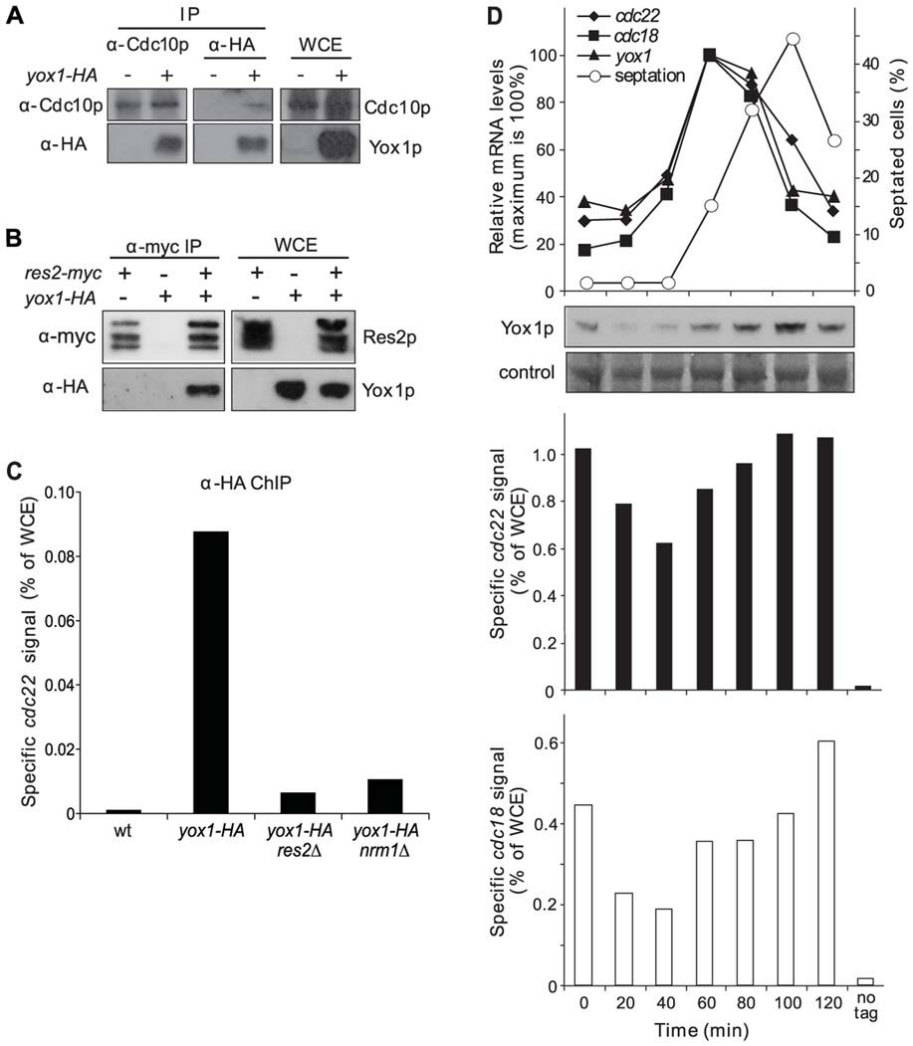
Yox1p binds via MBF to transcriptionally repressed MBF promoters. (A) Western blot analysis of extracts from strains carrying untagged or HA tagged Yox1p. Immune precipitates (anti-Cdc10p and anti-HA) and whole cell extract (WCE) were probed with anti- Cdc10p or anti-HA antibodies to detect Cdc10p and Yox1p, respectively. (B) Western blot analysis of extracts from strains carrying myc tagged Res2p, HA tagged Yox1p, or both. Anti-myc immune precipitates and whole cell extract (WCE) were probed with anti-myc or anti-HA antibodies to detect Res2p or Yox1p, respectively. (C) Bar graphs of PCR-amplified *cdc22* promoter fragments obtained from anti-HA ChIPs and quantified by qPCR as percentage of WCE signal. Signals are shown for cells without tagged Yox1p (wt) and for cells with HA tagged Yox1p in wild-type, *res2*Δ and *nrm1*Δ backgrounds. (D) Small wild-type cells were isolated by centrifugal elutriation and allowed to progress synchronously through the cell cycle with time points indicated at the bottom. Top graph: *cdc22*, *cdc18*, and *yox1* mRNA levels (100% is maximum) determined by RT qPCR, along with septation index (S-phase coincides with septation peak). Below top graph: Yox1p-HA protein levels are detected by anti-HA antibodies at the same time points, with amido black staining of the same membrane shown as loading control. Middle and bottom bar graphs: PCR-amplified *cdc22* and *cdc18* promoter fragments, respectively, generated from Yox1p-HA ChIPs and quantified by qPCR as percentage of WCE signal. Signals detected in an immunoprecipitation from an unsynchronised culture without tagged genes provides as a negative control (no tag).

No enrichment of homeodomain-related motifs was evident within the promoters of the shared Yox1p and MBF target genes (Matias Piipari, personal communication), raising the possibility that Yox1p binds to DNA via MBF. To test this hypothesis, we first checked whether Yox1p is part of the MBF complex by performing co-immunoprecipitation analyses. Anti-HA and anti-Cdc10p immune complexes prepared from cells expressing wild-type or HA-tagged Yox1p revealed an interaction between Cdc10p and Yox1p (Figure 2A). Moreover, anti-myc immuno complexes prepared from cells expressing Res2p-myc, Yox1p-HA or both showed an interaction between Res2p and Yox1p (Figure 2B) These results are confirmed by independent data from a recent mass spectrometry-based analysis of affinity-purified Res2p and Nrm1p complexes [20]. Based on these mass spectrometry data, Yox1p interacts with both Res2p and Nrm1p, with coverage of Yox1p by specific peptides being similar to the MBF component Cdc10p (data not shown). Together, these data indicate that Yox1p physically associates with the MBF complex and thus represents a new component of MBF.

To directly test whether Yox1p requires MBF to bind to MBF-regulated promoters, we used ChIP analysis to analyze Yox1p binding to the well-established MBF target gene *cdc22* (Figure 1D; [29]) in wild type, *res1*Δ and *nrm1*Δ cells expressing Yox1p-HA. This analysis revealed that Yox1p binding to the *cdc22* promoter depends on both of the MBF components tested, Res2p and Nrm1p (Figure 2C). We conclude that Yox1p can bind to MBF-regulated promoters only via intact MBF.

We next wondered about the dynamics of Yox1p levels and Yox1p binding to MBF-regulated promoters during the cell cycle. Consistent with *yox1* mRNA data (Figure 1A), the Yox1p levels were low during M/G1-phase but then strongly increased during S-phase (Figure 2D, top), peaking about 40 minutes after the peak of *yox1* mRNA levels. These data indicate that Yox1p is unstable and present at low levels during MBF-dependent transcription and at high levels when MBF-dependent transcription decreases. We also analyzed Yox1p binding to the two well-established MBF target genes *cdc22* [29] and *cdc18* [30] in cells synchronized by centrifugal elutriation. Consistent with the kinetics of its accumulation, Yox1p binding to the *cdc22* and *cdc18* promoters was observed throughout the cell cycle but substantially decreased at the time of transcriptional activation of the MBF targets, followed by an increase as cells progressed into S-phase when transcription became inactivated (Figure 2D). The Nrm1p co-repressor shows cell cycle-dependent binding profiles at MBF-regulated promoters similar to those observed here for Yox1p [20]. We conclude that dissociation of Yox1p from MBF-regulated promoters coincides with low Yox1p levels and with active transcription, whereas its subsequent re-association coincides with high Yox1p levels and with repressed transcription during the cell cycle.

### Yox1p Represses MBF Target Genes at the Transcriptional Level

**Figure 3:**
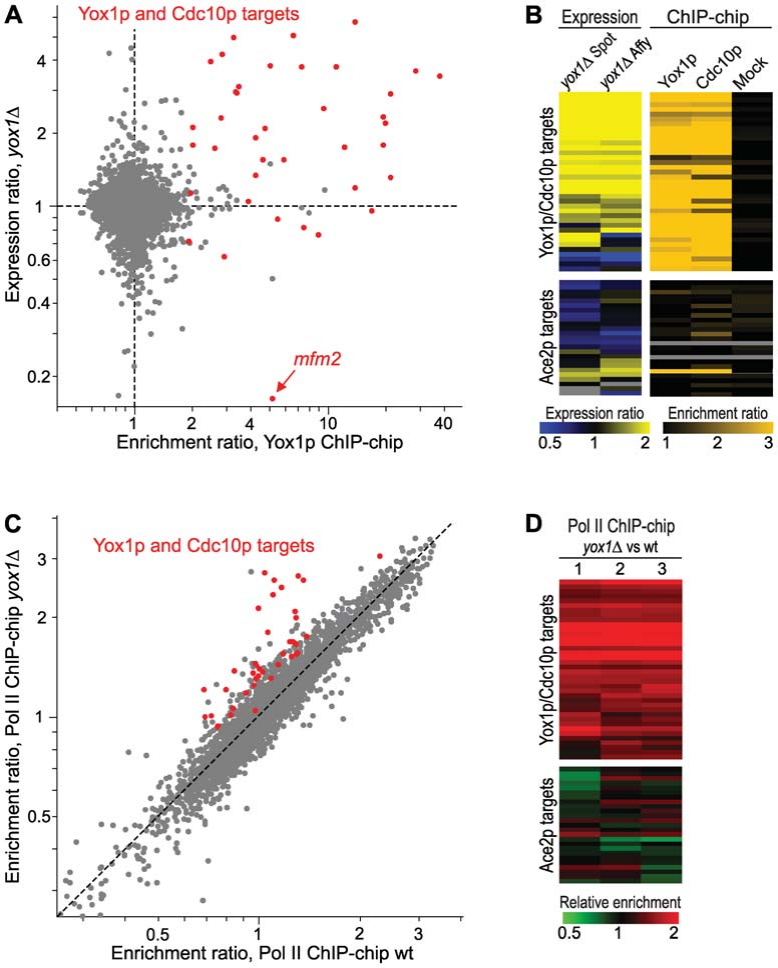
Yox1p inhibits transcription of target genes. (A) Yox1p and Cdc10p target genes tend to be more highly expressed in *yox1Δ* cells. Scatter plot showing gene expression relative to wild-type in *yox1Δ* mutant (mean data from three repeats on spotted arrays) *versus* the enrichment ratios upstream of genes in Yox1p ChIP-chip experiments (mean data from three repeats). The cell cycle-regulated target genes common to Yox1p and Cdc10p are highlighted in red, and *mfm2* is indicated with an arrow. (B) Cdc10p but not Ace2p target genes are highly expressed in *yox1Δ* cells. Hierarchical cluster analysis with rows representing Yox1p and Cdc10p target genes (top) or putative Ace2p-regulated genes (bottom; [14]). The two columns on the left represent expression profiling data of *yox1Δ versus* wild-type cells (average data of three repeats on spotted arrays and of two repeats on Affymetrix chips, respectively), with relative mRNA levels color-coded as indicated at the bottom. The three columns on the right represent ChIP-chip data (average data of two Cdc10p, five Yox1p, and two mock IPs, respectively), with the strength of enrichment color-coded as indicated at the bottom. Grey indicates missing data. (C) Yox1p and Cdc10p target genes tend to have higher Pol II occupancy in *yox1Δ* cells. Scatter plot showing the relative Pol II occupancy across genes in *yox1Δ* cells *versus* the relative Pol II occupancy across genes in wild-type cells (mean data from three repeats each). The target genes common to Yox1p and Cdc10p are highlighted in red as in (A). (D) Cdc10p but not Ace2p target genes show higher Pol II occupancy in *yox1Δ* relative to wild-type cells. Hierarchical cluster analysis with rows representing Yox1p/Cdc10p-regulated genes (top) or putative Ace2p-regulated genes (bottom; [14]). The three columns represent independent repeats of ChIP-chip experiments, with enrichment ratios in *yox1Δ* relative to wild-type cells color-coded as indicated at the bottom.

The binding data from Figure 2D suggested a negative role of Yox1p in MBF-regulated transcription. To obtain direct insight into Yox1p function at target genes, we deleted the *yox1* gene and compared global gene expression levels in *yox1Δ versus* wild-type cells using both spotted DNA microarrays and Affymetrix chips (Table S1). The cell cycle-regulated target genes bound by both Yox1p and Cdc10p tended to be more highly expressed in *yox1Δ* than in wild-type cells (Figure 3A,B). To test whether the higher expression of Yox1p/Cdc10p target genes might reflect an indirect effect caused by any cell-cycle delay in the *yox1Δ* cells, we also analyzed the expression profiles of Ace2p target genes, whose expression peaks coincide with the MBF target genes [14],[31]. In contrast to the Yox1p/Cdc10p target genes, the Ace2p target genes were generally not expressed at higher levels in the *yox1Δ* cells and were not bound by Yox1p or Cdc10p (Figure 3B). An exception was the putative Ace2p target gene *klp8*, encoding a kinesin-like protein, which was bound by both Yox1p and Cdc10p and induced in *yox1Δ* cells, suggesting that *klp8* is regulated by both Ace2p and MBF. Taken together, the expression signature of *yox1Δ* cells indicates that Yox1p inhibits expression of its target genes.

To directly test whether inhibition of gene expression by Yox1p occurs at the transcriptional level, we determined the global RNA Polymerase II (Pol II) occupancy across coding regions in both *yox1Δ* and wild-type cells, which provides an estimate of transcriptional efficiency [32]. The Yox1p/Cdc10p target genes showed consistently higher Pol II occupancy in *yox1Δ* than in wild-type cells (Figure 3C,D), whereas the Ace2p target genes showed similar Pol II occupancy in the two strains (Figure 3D). We conclude that Yox1p, unlike Cdc10p, generally plays a negative role in the transcription of its target genes.

A few genes, however, seem to be positively regulated by Yox1p (Figure 3A,B). An example is *mfm2* (Figure 3A), encoding a precursor for the M-factor peptide (a mating pheromone) [33], and the neighbouring *SPAC513.04*, a sequence orphan that is divergently transcribed from *mfm2*. Another example is *map1*, encoding a MADS-box transcription factor involved in the transcriptional response during mating [34]. Among these genes, only *mfm2* is periodically expressed during the cell cycle [35]. Notably, *yox1* expression is induced during mating and early meiosis [36],[37], and *yox1Δ* cells seemed to exhibit mating defects (unpublished observations), similar to what has been observed for *res2*Δ [38] and *nrm1*Δ cells (unpublished observations). Together, these findings raise the intriguing possibility that these MBF components also play a positive role during cell mating, which will require further work to unravel.

### Yox1p Is Required for Cell-Cycle Regulation of MBF Target Genes

**Figure 4:**
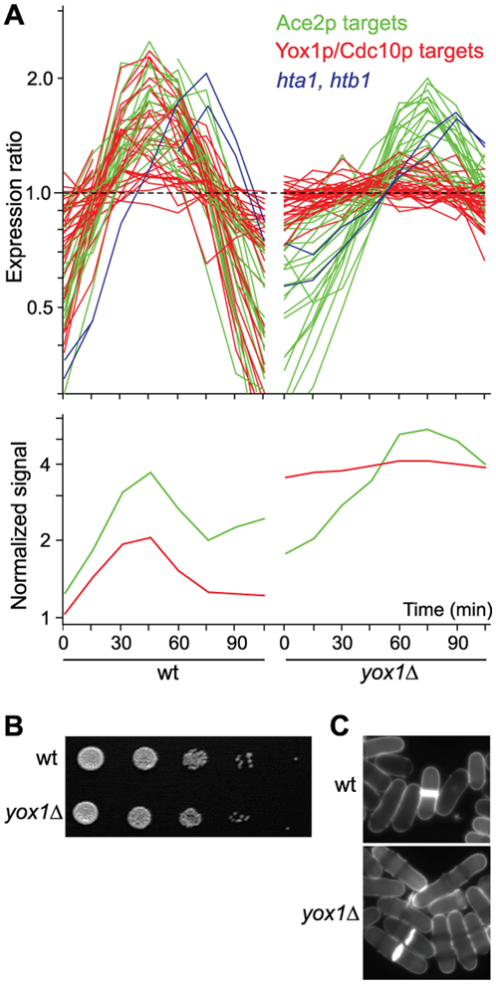
Cell-cycle defects in *yox1Δ* cells. (A) Yox1p/Cdc10p target genes are not cell cycle-regulated in *yox1Δ* cells. Top graph: Two cell-cycle timecourse experiments of wild-type cells (left; [14]) and *yox1Δ* cells (right) synchronized by centrifugal elutriation, showing the expression profiles of cell cycle-regulated target genes common to Yox1p and Cdc10p (red) and putative Ace2p target genes (green; [14]). The histone genes *hta1* and *htb1* are highlighted in blue. The expression profiles were measured using spotted arrays and normalized such that the median expression ratios are equal to 1 for each timecourse. Bottom graph: As top graph but showing the mean absolute signal intensities, normalized to 50^th^ percentile of measurements from each array, for Ace2p (green) and for Yox1p/Cdc10p (red) targets. Note that the timepoints of wild-type and *yox1Δ* experiments are not normalized relative to the cell cycle, and absolute times cannot be directly compared. (B) Normal growth of *yox1Δ* cells. 10-fold serial dilutions of wild-type (top) and *yox1Δ* (bottom) cells spotted on rich media. (C) Elongated phenotype in *yox1Δ* cells. Wild-type (top) and *yox1Δ* (bottom) cells were stained with calcofluor to highlight cell wall and division septa.

Given that Yox1p bound to MBF target promoters in a cell cycle-dependent manner and was required for transcriptional repression of MBF-regulated genes, we next examined the role of Yox1p in periodic cell-cycle transcription of the Yox1p/Cdc10p target genes. To this end, we synchronized *yox1Δ* cells using centrifugal elutriation and compared microarray expression profiles with those of wild-type cells. In wild type cells, the Yox1p/Cdc10p and Ace2p target genes all peak during S-phase/cell division (Figure 4A; [1]). In *yox1Δ* cells, on the other hand, the Yox1p/Cdc10p targets showed little or no cell-cycle regulation, whereas the cell-cycle regulation of Ace2p targets was not affected (Figure 4A). Exceptions were the histone genes *hta1* and *htb1*, which peak later than the other Yox1p/Cdc10p targets and maintained some cell-cycle regulated expression in *yox1Δ* cells, albeit with lowered amplitude (Figure 4A). We also analyzed the normalized signal intensities to estimate absolute expression levels, which revealed that the Yox1p/Cdc10p target genes, but not the Ace2p target genes, were continually higher expressed during the cell cycle in the absence of Yox1p (Fig. 4A, lower graph). Taken together, we conclude that cell-cycle regulated transcription of Yox1p/Cdc10p target genes is highly deficient in *yox1Δ* cells, reflecting that these genes are no longer down-regulated after S-phase.

To check whether increased transcription and absence of cell-cycle regulation of Yox1p/Cdc10p target genes leads to any obvious defects, we also analyzed the phenotype of the *yox1Δ* mutant at the cellular level. *yox1Δ* cells were viable and did not show any overall growth defect (Figure 4B). The septation index was marginally higher in *yox1Δ* compared to wild-type cells (10.4% *vs* 9.4%), suggesting a slight delay in cytokinesis and/or cell separation. The *yox1Δ* cells were about 14% longer on average than wild-type cells during septation (17.4 µm *vs* 15.3 µm) (Figure 4C). While fewer than 2% of wild-type cells were longer than 18 µm during septation, 31% of *yox1Δ* cells exceeded this length. A few *yox1Δ* cells (∼5%) also contained excessive vacuoles (not shown). FACS analysis showed that the bulk of *yox1Δ* cells were in G2-phase as is the case for wild-type cells (data not shown). Together with the elongated phenotype, this finding suggests that *yox1Δ* cells are somewhat compromised in cell-cycle progression after S-phase.

### Common and Specific Targets of Cdc10p and Yox1p

**Figure 5:**
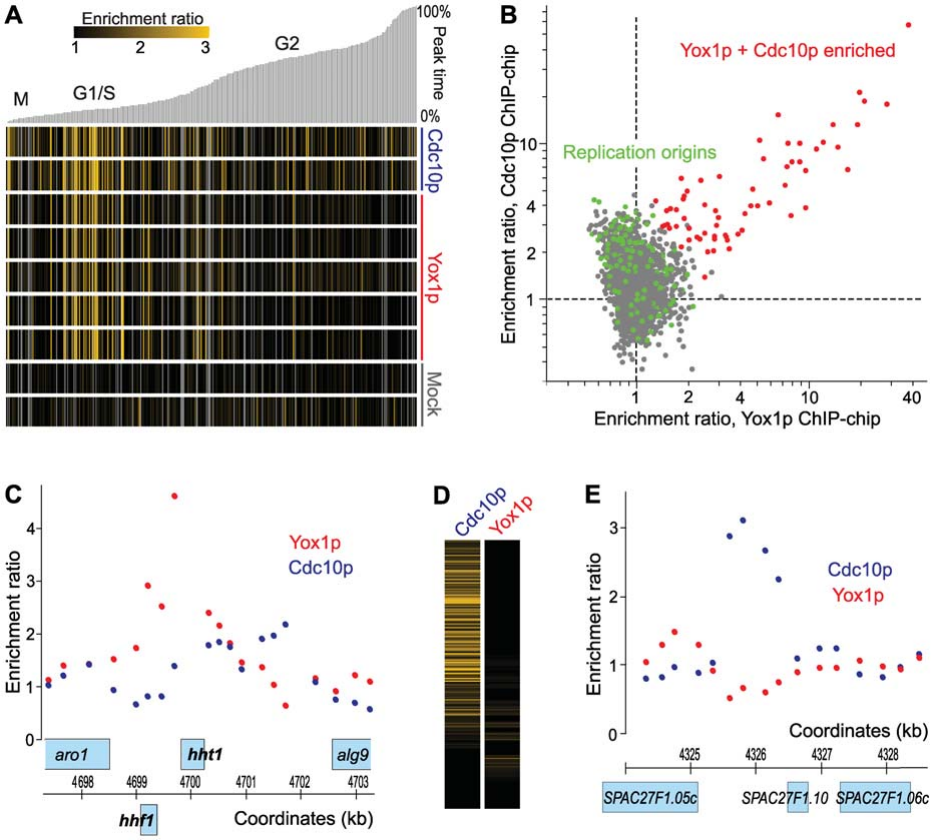
Common and specific Cdc10p and Yox1p targets. (A) Cdc10p and Yox1p target genes predominantly peak in expression around G1/S. The top-500 cell cycle–regulated genes were ordered in columns by their peak expression times [35] with 0% and 100% of cell-cycle time set around the G2/M transition. Relative peak times are indicated on top along with approximate cell-cycle phases. The rows (from top to bottom) correspond to two independent repeats of Cdc10p ChIP-chip, five independent repeats of Yox1p ChIP-chip, and two independent repeats of mock IPs. The orange shading reflects the strength of relative enrichment as indicated in legend (top left). (B) Comparison of Cdc10p and Yox1p targets. Scatter plot showing the enrichment ratios upstream of genes in Cdc10p ChIP-chip experiments (mean data from three repeats) *versus* the enrichment ratios upstream of genes in Yox1p ChIP-chip experiments (mean data from three repeats). The target genes common to Yox1p and Cdc10p are highlighted in red, and the genes flanking replication origins [40] are highlighted in green, many of which are specifically enriched in Cdc10p ChIP-chip. (C) The divergently transcribed histone H3 and H4 genes (*hht1* and *hhf1*, respectively) show enrichment of Yox1p (red), but not of Cdc10p (blue), in their shared promoter region. Example data from one repeat each are shown. Transcription of the three upper genes is from left to right (forward strand), while *hhf1* is transcribed from right to left (reverse strand). Chromosome 1 coordinates are indicated in kb. (D) Cdc10p, but not Yox1p, frequently binds close to replication origins. Hierarchical cluster analysis with rows representing 445 genes flanking origins of replication [40] and columns representing Cdc10p ChIP-chip (left, average of all experiments) and Yox1p ChIP-chip (right, average of all experiments). The strength of relative enrichment is indicated by orange shading as in (A). (E) The intergenic region between the genes *SPAC27F1.05c* and *SPAC27F1.10* contains an origin of replication and shows enrichment of Cdc10p (blue), but not of Yox1p (red). Neither of these genes is cell cycle–regulated. Example data from one repeat each are shown. Transcription of the three genes is from right to left (reverse strand). Chromosome 1 coordinates are indicated in kb.

Our ChIP-chip experiments also provided a global overview of genomic regions directly bound by Cdc10p and Yox1p. The enrichment ratios of all ChIP-chip experiments are provided in Table S2. Among cell cycle-regulated genes, most Cdc10p and Yox1p target genes peaked in transcript levels around G1/S (Figure 5A), consistent with the results described above. Cdc10p also seemed to weakly bind to some cell-cycle-regulated genes outside of G1/S phase (Figure 5A), raising the possibility that this factor has additional regulatory roles, perhaps in combination with other cell-cycle transcription factors.

The promoters of 76 genes were substantially and consistently enriched in both Cdc10p and Yox1p ChIPs, including the 19 previously discussed MBF target genes (Fig 5A,B; Table S3). These genes also significantly overlapped with lists of putative Cdc10p, Res1p, and Rep2p target genes, which were obtained from independent ChIP-chip experiments using custom-spotted intergenic arrays (*P* = 3e-26 to 8e-16). Both Yox1p and Cdc10p bound to the promoter between the divergently transcribed histone genes *hta1* and *htb1* (H2Aα and H2Bα, respectively) and to *pht1* (H2A variant), while Yox1p was also bound to the promoter between the divergently transcribed histone genes *hht1* and *hhf1* (H3 and H4, respectively) (Figure 5C). In total, 40 of the 76 common target genes were among the top-500 cell cycle-regulated genes [35]. The promoters of these 40 genes were significantly enriched for different versions of MCB motifs (Matias Piipari, personal communication). These experiments thus uncovered additional MBF target genes and, as expected, many of these genes are involved in DNA replication and repair (Table S3). Among the 36 genes enriched in Cdc10p and Yox1p ChIPs that were not cell cycle-regulated, 10 genes were also enriched in the independent Cdc10p, Res1p and Rep2p ChIP-chip experiments using intergenic arrays (*P* ∼2e-17), although 6 of these genes are divergently expressed from neighboring Cdc10p target genes and the enrichment may therefore reflect binding at the neighboring promoter. The four exceptions are *mei3*, *gdi1*, *spt6*, and a putative non-coding RNA, *SPNCRNA.93*. Visual inspection of their expression profiles during the cell cycle, however, confirmed that these genes do not seem to be periodically transcribed.

The experiments shown in Figures 3 and 4A also helped to identify further Yox1p/Cdc10p targets, for which the ChIP-chip experiments were not conclusive. For example, *SPAC17H9.18c* and *ctp1* were more highly expressed and showed no cell-cycle regulation in *yox1Δ* cells, indicating that they are true Yox1p targets. We have therefore added these genes to Table S3. *SPBPB2B2.19c, SPAC750.05c* and *rep2*, on the other hand, were not expressed at higher levels and showed strong cell-cycle regulation in *yox1Δ* cells, indicating that they are likely not Yox1p targets, consistent with the ChIP-chip data. Taken together, we conclude that the most strongly bound promoters are highly coherent between Cdc10p and Yox1p and provide a global survey of MBF target genes.

Yox1p, but not Cdc10p, also showed consistent, albeit weak, enrichment in association with genes located close to telomeres. Further work will be required to establish whether Yox1p plays an additional role at these telomere-associated genes. Cdc10p, on the other hand, showed substantial and specific enrichment upstream of>200 additional genes. Only 12 of these Cdc10p-specific target genes were among the top-500 cell cycle-regulated genes [35]. Among these genes were four 5S ribosomal RNAs (*SPRRNA.19, SPRRNA.20, SPRRNA.34*, and *SPRRNA.38*), which is consistent with the finding that Cdc10p binds to Pol5p that is required for rRNA transcription [39]. Intriguingly, the Cdc10p-specific target genes were significantly enriched for genes flanking mitotic origins of replication (Figure 5B; *P* = 1e-15) [40]. Most of the genes flanking origins of replication were associated to some extend with Cdc10p (Figure 5D). Unlike for MBF target genes, the Cdc10p enrichment associated with replication origins peaked near the centre of intergenic regions in some cases (Figure 5E), although the array resolution was generally insufficient to distinguish binding patterns relative to genes. These data suggest that Cdc10p has an affinity for replication origins.

## Discussion

In this study, we provide a systematic analysis of genome-wide binding sites for Yox1p and Cdc10p and show that these factors share more than 40 target genes that are transcriptionally induced during G1/S and are repressed during the other cell-cycle phases. Furthermore, we establish Yox1p as a new regulatory MBF component with a critical role in the MBF-dependent transcriptional program. In agreement with our observation that Yox1p does not directly bind to MBF-regulated promoters but via MBF, no enrichment of homeodomain-related motifs is evident within the promoters of the shared target genes (Matias Piipari, personal communication) and the binding sites of Yox1p and Cdc10p coincide (Figure 1D). The Yox1p and Cdc10p target genes show higher transcript levels and higher Pol II occupancy in the absence of Yox1p, and they also require Yox1p for their periodic down-regulation during the cell cycle. Consistently, Yox1p accumulates as cells progress into S-phase, and its binding to promoters peaks when MBF-dependent transcription is repressed. Yox1p therefore plays a negative role for the transcription of its target genes outside of G1/S.

**Figure 6:**
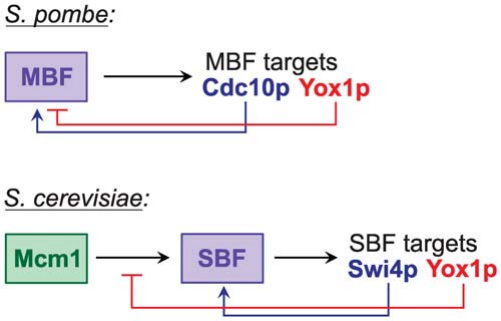
Yox1p-based negative feedback loops in fission and budding yeast. Positive and negative regulation is indicated by arrows and bars, respectively. See main text for detailed comparison of regulatory circuits.

Yox1p provides a negative feedback: it is transcriptionally activated by MBF and in turn binds to MBF and transcriptionally represses the MBF target genes (including *yox1* itself). This regulatory circuit has parallels but also intriguing differences to the circuit involving *S. cerevisiae* Yox1p (Figure 6). In *S. cerevisiae*, the MADS-box factor Mcm1p is involved in transcriptional activation of *SWI4* (similar to *cdc10*) and *CLN3* genes [41]–[43], thus promoting activation of the SBF complex, which is orthologous to *S. pombe* MBF [1]. SBF then activates transcription of several targets including *S. cerevisiae YOX1* [27],[28],[44]. Yox1p in turn binds next to the Mcm1p target sequences and inhibits Mcm1p-dependent transcription and thus also the downstream SBF-dependent transcription [26]. In both yeasts, the MBF/SBF complexes therefore transcriptionally activate a negative feedback via Yox1p, which either directly (*S. pombe*) or indirectly (*S. cerevisiae*) inhibits MBF/SBF-mediated transcription (Figure 6). Budding yeast contains a Yox1p paralog, Yhp1p, which is not cell cycle-regulated but also acts as a repressor of Mcm1p-dependent transcription [26]. No such paralog is present in fission yeast.

Two negative feedback loops have previously been reported to constrain transcription to G1/S by promoting timely repression of MBF-dependent genes: 1) the cyclin Cig2p, whose gene is activated by MBF, in turn inhibits MBF activity in G2-phase by phosphorylating Res1p [19]; and 2) Nrm1p, whose gene is also activated by MBF, in turn acts as a transcriptional co-repressor for MBF-dependent genes much like Yox1p [20],[21]. Consistently, our data show that both Cdc10p and Yox1p bind to *cig2* and *nrm1* promoters. Notably, genetic perturbation of *yox1, nrm1*, or *cig2* leads to increased MBF-dependent transcription, although *cig2* mutants show residual transcriptional repression of MBF targets outside of G1/S. All proteins seem therefore to be required, but are not sufficient, to repress MBF transcription outside of G1/S. This repression is not essential in rapidly growing cells: *cig2Δ, nrm1Δ* and *yox1Δ* cells are viable, although all deletion mutants show some cell elongation phenotype ([45]; unpublished data; this study). It is possible that the repression becomes more critical under specific conditions such as inhibited proliferation during nutrient limitation. Notably, deletion of *yox1* has been reported to lead to cadmium sensitivity [46].

We propose that there are two independent negative feedback mechanisms that tune down MBF-regulated transcription, one involving Cig2p and the other involving both Yox1p and Nrm1p. The following evidence indicates that Yox1p and Nrm1p act in the same pathway. The increase of MBF-dependent transcript levels in unsynchronized *yox1*Δ cells (Figure 3) is similar to that observed in *nrm1*Δ cells (unpublished data). Moreover, a *yox1*Δ *nrm1*Δ double deletion mutant does not show any increased MBF-regulated transcript levels compared to either single deletion alone (unpublished data). Both *yox1*Δ and *nrm1*Δ cells also show very similar phenotypes with respect to cell elongation, mating defects, and synthetic lethality with *cdc25-22* (this study; unpublished data). Furthermore, binding of Yox1p to MBF-regulated promoters depends on Nrm1p (Figure 2C). Together, these findings suggest that Yox1p and Nrm1p operate in the same negative feedback loop in which the role of Yox1p in transcriptional repression depends on Nrm1p. Whether Nrm1p depends on Yox1p for its association with MBF or is a connector protein required for Yox1p-dependent repression of MBF-regulated transcription remains to be determined.

Besides the negative feedback loop based on Yox1p, both yeasts also seem to apply positive feedback loops based on the SBF-regulated *SWI4* in budding yeast [41],[47] or the MBF-regulated gene *cdc10* in fission yeast ([14]; this study) (Figure 6), although Cdc10p may have both positive and negative roles in MBF-mediated transcription [24],[48].

Cells arrested in S-phase with incompletely replicated DNA show persistent expression of MBF-dependent genes [14],[15], which depends on the DNA replication checkpoint pathway and is required for cell survival [23],[24]. Recent data indicate that the DNA replication checkpoint maintains MBF-dependent transcription by inactivating the Nrm1p repressor [20] and by directly regulating Cdc10p [24]. It is possible that Yox1p is also involved in regulating the persistent MBF-dependent transcription during the checkpoint response or in mediating checkpoint recovery.

Yox1p also binds to genes encoding all four canonical histones (*hht1, hhf1, hta1, htb1*) and the histone variant *pht1*, while Cdc10p binds only to *hta1*, *htb1*, and *pht1*. It therefore seems that Yox1p can also bind independently of MBF to some target genes that are not regulated by MBF (Figure 5C). With the exception of *pht1*, which is ∼2-fold higher expressed and shows no periodic transcription in *yox1Δ* cells, we could detect only subtle, if any, effects on histone gene expression in the absence of Yox1p, which may therefore only have a marginal role in the poorly understood control of these highly regulated genes. *S. cerevisiae* Yox1p also binds to the genes encoding histones H3 and H4, but not to H2A and H2B, and it has been suggested to play a role in their transcriptional regulation [27]. In *S. pombe*, the GATA-type transcription factor Ams2p binds to the promoter regions of core histone genes and is necessary for their transcriptional activation [49]. Moreover, the HIRA-like protein Hip1p is involved in repressing histone genes outside of S-phase [50]. Intriguingly, *hip1* is a Cdc10p (but not a Yox1p) target according to our data, although *hip1* transcript levels do not seem to be cell cycle-regulated. It is possible that this regulatory interaction becomes more important during specialized conditions such as starvation or meiosis.

Cdc10p showed more promiscuous binding across the genome than Yox1p. Many of the additional target regions coincided with origins of mitotic replication [40], and most replication origins were associated with Cdc10p (Figure 5D). The genes flanking replication origins are not enriched for MBF- or cell cycle-regulated genes, and these regions do not seem to be enriched for MCB elements recognized by MBF (Matias Piipari, personal communication). It is possible that Cdc10p simply binds to these chromatin regions because they are depleted of nucleosomes and thus accessible [51]. However, this finding also raises the intriguing possibility that Cdc10p, either alone or in complex with other proteins, is directly involved in controlling replication origins, in addition to its role in transcriptionally regulating genes functioning during replication. Cdc10p would certainly be present at the right places and at the right time during the cell cycle for such a role. Notably, *Drosophila* dE2F1, the functional equivalent of Cdc10p, also binds to origins of replication [52]. This study indicates that dE2F1 functions at replication origins to limit DNA replication through its interaction with the origin recognition complex [52]. Future research will establish whether Cdc10p plays an active role at replication origins and is directly involved in controlling DNA replication.

In conclusion, we have uncovered an additional regulatory layer, based on the homeodomain protein Yox1p, to switch-off MBF-dependent transcription during S-phase progression. Similar roles have been identified for Cig2p and Nrm1p. The requirement for these multiple, non-redundant negative feedback mechanisms is striking. Although potentially rendering down-regulation of G1/S transcripts during S-phase less robust, this system might provide an additional mechanism by which checkpoint activation could override the regular periodic transcriptional program, by directly regulating Yox1p (as has been shown for Nrm1p and Cdc10p [20],[24]) and may thus reflect the importance of timely and robust activation of G1/S transcription in response to problems with DNA replication. It will be important to tease out how the different regulatory layers are coordinated with each other during regular cell proliferation, during meiotic differentiation, and in response to perturbation of DNA replication.

## Materials and Methods

### Yeast Strains, Experimental Conditions, and Phenotyping

The *yox1* gene was deleted in a diploid background made from strains JB5 (*ade6-210 h^+^*) and JB6 (*ade6-216 h^−^*) using the *KanMX6* marker with a PCR-based approach [53], resulting in strain JB361 (*yox1Δ::kanMX6 ade6^−^ h^+^*). *yox1* was C-terminally tagged with a *3HA* epitope in strain JB22 (*972 h^−^*) with the PCR-based approach [53] and verified by western blot, resulting in strain JB644B (*yox1-3HA::kanMX6 h^−^*). All strains were grown in rich medium (YE+supplements) at 32°C. Centrifugal elutriation was performed as described [14]. As a reference for all time points, we used unsynchronized cells taken from the same culture before elutriation.

For septation index and cell length measurements, duplicate cell populations at a density of ∼5×10^6^ cells/ml were fixed in 10% formaldehyde for 15 minutes, washed three times in 1 x PBS and stored at 4°C. Calcofluor (50 µg/ml; Polysciences) was used to stain the septa and cell wall. Images were captured using a Hamamatsu digital camera C4742-95 fitted to a Zeiss Axioskop microscope with plan-Apochromat 63×1.25 oil objective and recorded using OpenLab 3.4 software (Improvision), downloaded to either Microsoft Excel for analysis or to Adobe Photoshop 7. To determine the septation index, the amount of fully septated cells was calculated as a percentage of the total population (n>700 each strain). The mean maximal cell length was calculated from measurements of fully septated cells (n>300 each strain). For FACS analysis, cells were fixed in 70% ethanol at ∼5×10^6^ cells/ml, washed with 10 mM EDTA, and treated with 0.1 mg RNAseA overnight at 37°C. DNA was subsequently stained with 4 µg/ml propidium iodide, and cells were sonicated prior to FACS analysis. 10,000 cells, in duplicate samples, were analysed using a Cyan ADP Flow Cytometry system (Beckman-Coulter).

### Chromatin Immunoprecipitation (ChIP)

Our protocol was adapted from [54]. For each IP, 50 ml of exponentially growing cells (titer: 6×10^6^ cells/ml) were fixed with 1% formaldehyde for 30 min, and the reaction was stopped by adding 2.5 ml of 2.5M glycine. Cells were mechanically broken with glass beads (BioSpec) in lysis buffer containing protease inhibitors by vortexing 2×13 sec. Subsequently, the lysates were sonicated in a Bioruptor (Diagenode) for 3×5 min, with 30 sec ON and 30 sec OFF. A 50 µl aliquot per sample was immediately withdrawn and saved as input. Protein DNA-complexes were immunoprecipitated with 5 µg of antibody by incubating overnight at 4°C with 50 µl protein A Sepharose beads (Amersham). We used polyclonal anti-Cdc10p antibody (a kind gift of Jérome Wuarin), anti-HA antibody (Abcam 9110), and anti-Pol II antibody (Abcam 5408) for Cdc10p, Yox1p, and Pol II IPs, respectively. The immunoprecipitated material was washed and eluted twice in TES, and the samples were then treated with Proteinase K and de-crosslinked for 5 hours at 65°C. Following de-crosslinking, RNA was digested for 1 h at 37°C. DNA was extracted once with phenol/chloroform/isoamyl alcohol and precipitated with 3M NaAc and 100% ethanol. Precipitated DNA pellets were air-dried and resuspended in 20 µl double-distilled water. ChIP analysis followed by qPCR with the synchronised strain JB644B (*yox1-HA*) was performed as described [55].

### ChIP-chip Assay and Data Analysis

For Cdc10p, we performed two independent biological ChIP-chip repeats plus one technical repeat with dye swaps using wild-type strain JB22 (972 *h^−^*). For Yox1p, we performed five independent biological ChIP-chip repeats plus one technical repeat with strain 644B (*yox1-HA*), using dye swaps for one biological and the technical repeat. For Pol II, we performed three independent biological ChIP-chip repeats with strain 644B (*yox1-HA*) and three independent biological repeats with strain JB361 (*yox1Δ*). Mock IPs were performed from two independent biological repeats with strain JB361.

For the Agilent platform, input and immunoprecipitated (and mock) material were amplified by random PCR amplification as described [56]. For labelling of amplified DNA, 500 ng per sample was used for incorporation of Cy3 (input) and Cy5 (IP material) d-CTP nucleotides as recommended in the genomic DNA Bioprime labelling kit (Invitrogen). For dye swap experiments, Cy3 was used for IP material and Cy5 for input material. Hybridization and washes were carried out according to the manufacturer's instructions, and guidelines for the 4x44K Chip-on-chip whole genome DNA microarray platform (Agilent). For the custom-spotted intergenic arrays, the IP DNA was not amplified but directly labelled with the Bioprime kit and hybridized against a directly labelled mock IP performed with rabbit IgG. Hybridization and washes were then performed as previously described [40].

The Agilent arrays were scanned in a GenePix 4000B laser scanner at 5 µm resolution, and the acquired fluorescent signals were subsequently processed for analysis with GenePix Pro 6.0 software (Axon instruments). The data were then imported in Bioconductor version 2.6.1, and systematic or array bias was removed by the variance stabilization algorithm (vsn) [57]. Briefly, each column (Cy3 and Cy5) on the array was calibrated by an affine transformation and then the data were transformed by a glog2 variance stabilizing transformation. After normalization, signal ratios were obtained for each array element. In order to determine enrichment at promoter regions, we calculated the mean intensity of all probes located within 1000 bp upstream of each open reading frame. For the Pol II ChIP-chip experiments, the mean intensities of probes were calculated for all coding regions.

In order to determine statistically significant enrichment over a promoter or coding region, we applied SAM statistics (Significance Analysis of Microarrays) [58]. For the Yox1p ChIP-chip experiments, we compared six independent repeats (with one repeat performed as technical repeats) and two independent mock IPs. We determined a conservative list of significantly enriched promoters using 0% FDR (false discovery rate). For the Cdc10p ChIP-chip experiments, we ranked the promoter ratios from all three repeats (2 biological, 1 technical) and used a conservative cut-off of 2-fold for enrichment relative to the input. Overlaps between functional gene lists were determined in GeneSpring GX 7.3 (Agilent), using a standard Fisher's exact test, and the p-values were adjusted with a Bonferroni multiple testing correction. The ChIP-chip data are available from ArrayExpress under the accession number E-TABM-647.

### Co-Immunoprecipitation

Anti-HA, anti-myc and anti-Cdc10p immunoprecipitations were carried out using TAP purification buffers [59]. Immunoprecipitated proteins were resolved by 10% SDS-PAGE. Anti-HA, anti-myc and anti-Cdc10p antibodies as described above were used to detect HA-tagged proteins and endogenous Cdc10p, respectively.

### Real-Time PCR and RT PCR

Total RNA was isolated using the Rneasy Plus kit (Qiagen). The iQ SYBR Green supermix (Biorad) was used for quantitative PCR on ChIP samples, and the iScript One-Step RT-PCR kit with SYBR Green (Biorad) was used for RT-PCR experiments. Reactions were run on the Chromo-4 Real-Time PCR Detector (Biorad) using standard PCR and RT/PCR conditions. Data were analyzed using MJ Opticon Monitor Analysis Software 3.0.

### Microarray Expression Profiling

RNA was extracted from logarithmically growing cultures as described [60]. For the Affymetrix platform, two technical repeats from wild-type strain JB22 (*972 h^−^*) and two independent biological repeats, one with an additional technical repeat, from strain JB361 (*yox1*Δ) were used to extract 5 µg of RNA each. The RNA was labelled using the standard Affymetrix GeneChip eukaryotic hybridization protocols, and all samples were hybridised on the Affymetrix Yeast 2.0 GeneChip arrays. Scanning was performed on a GeneChip Scanner 3000. Data were extracted and normalised in Bioconductor version 2.6.1 using the GCRMA package [61]. Ratios were obtained by dividing the probe values for each *yox1*Δ repeat over the averaged probe values from the two wild-type repeats.

For our custom-spotted arrays, RNA extracted from three independent biological repeats each from strain JB361 (*yox1*Δ) and strain JB6 (*ade-216 h^−^*) was labelled by direct incorporation of Cy3 and Cy5 d-CTP as instructed in the Superscript reverse transcriptase kit (Invitrogen). The cDNA was then hybridized on custom-spotted microarrays containing the known and predicted *S. pombe* genes [60]. Microarrays were washed and scanned using a GenePix 4000B laser scanner, and the acquired fluorescent signals were subsequently processed for analysis with GenePix Pro 6.0 software (Axon instruments). After removing flawed spots, the data were normalised using a Perl script as described [60]. The transformed expression ratios were then imported into GeneSpring GX 7.3 for further data analyses. The expression profiling data are available from ArrayExpress under the accession number E-TABM-646.

## Supporting Information

Table S1 Expression ratios of *yox1Δ* relative to wild-type cells for all genes from three custom-spotted arrays and two Affymetrix chips
(0.95 MB XLS)

Table S2 Enrichment ratios of all genes in two Cdc10p, five Yox1p, and two mock ChIP-chip experiments
(1.31 MB XLS)

Table S3 Genes enriched in both Yox1p and Cdc10p ChIP-chip
(0.03 MB XLS)
